# Supplementary material for: A neural implementation model of feedback-based motor learning
Source: Nat Commun. 2025 Feb 20;16:1805. doi: 10.1038/s41467-024-54738-5 (PMC11842561; doi:10.1038/s41467-024-54738-5)
Supplement: Supplementary file 1 — Supplementary Information [file 41467_2024_54738_MOESM1_ESM.pdf]

Supplementary Figures

Supplementary Figure 1. Hypothesis.

Supplementary Figure 2. Initial training protocol.

Supplementary Figure 3. Recurrent connectivity enables stable control with delayed feedback.

Supplementary Figure 4. Both feedback-based motor control and adaptation can be achieved with sparse feedback signals which are small compared to recurrent signals.

Supplementary Figure 5. Effective feedback-based motor control and adaptation can both be achieved with sparse feedback for varied degrees of recurrent connectivity.

Supplementary Figure 6. Control and adaptation can be achieved with noisy signals and less localised plasticity rule.

Supplementary Figure 7. Online motor control and feedback-based learning can be achieved for both higher-dimensional joint position and velocity feedback, and for endpoint instead of trajectory control.

Supplementary Figure 8. Activity changes during VR for monkey PMd and M1.

Supplementary Figure 9. Extended analyses for experiments presented in Figure 5.

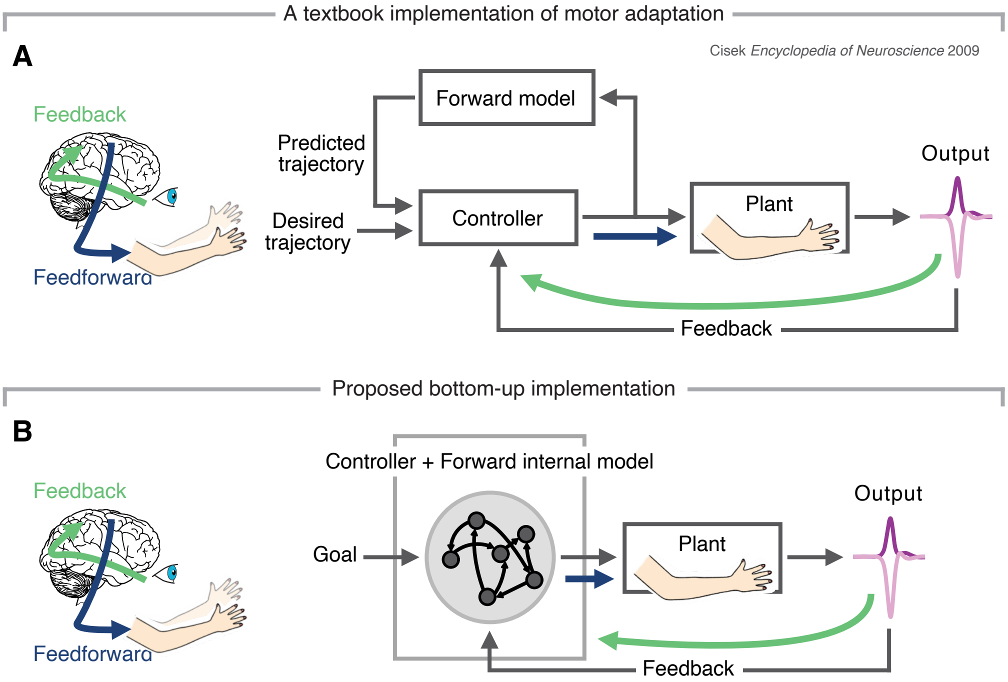

Supplementary Figure 1: Hypothesis. Comparison between a classic model of motor adaptation in which a forward internal model is updated to drive adaptation (adapted from Ref. 1) (A), and the model proposed in this manuscript (B). The brain image is from NIAID Visual Medical Arts. 06/27/2024. Lateral Brain. NIAID NIH BIOART Source. https://bioart.niaid.nih.gov/bioart/60

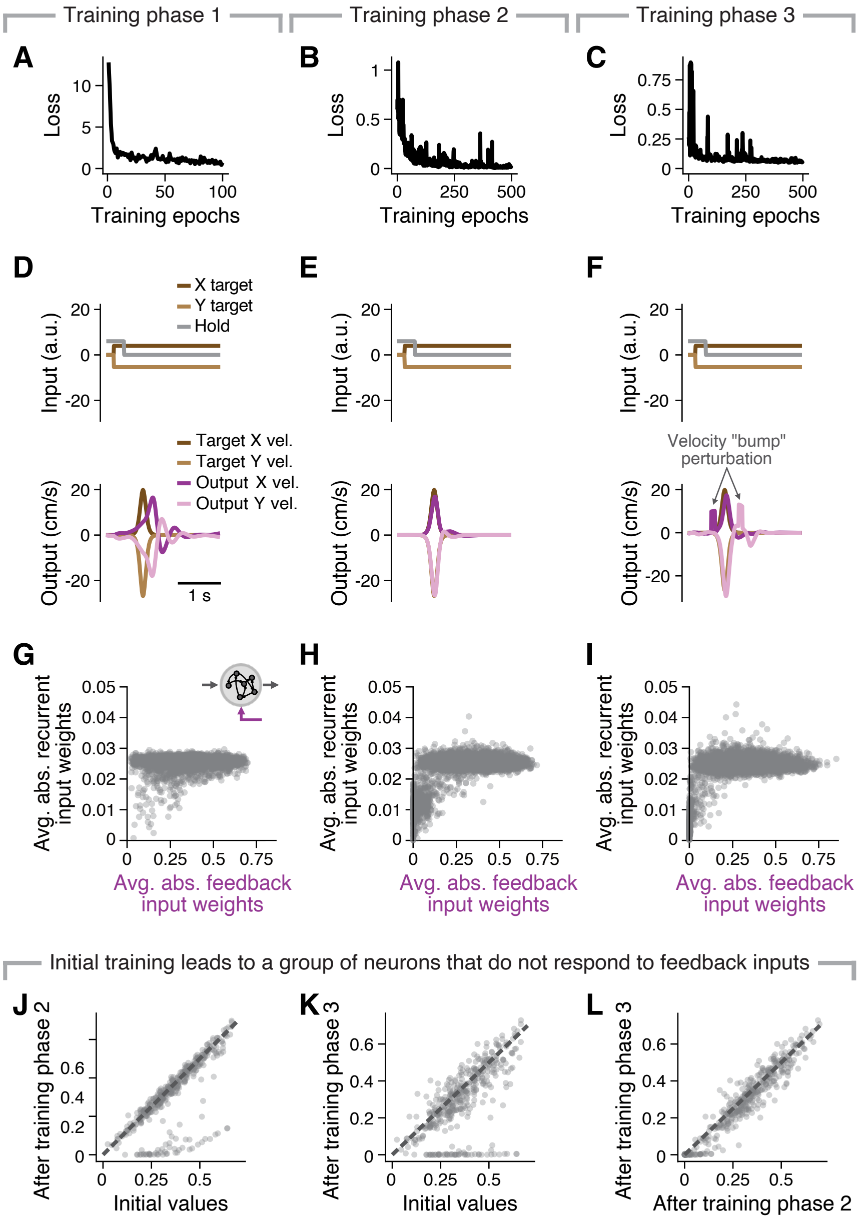

Supplementary Figure 2: Initial training protocol. The initial training of the RNN is divided into three phases. In the first phase, we kept the feedback weights W^{fb} fixed and at small initial values (A,D). In the second phase, we lifted that constraint, and all model parameters became plastic (B,E). In the third phase, we introduced random velocity perturbations in 75 % of trials (C,F). **A-C.** Training loss for each of the three training phases. **D-F.** Input (top) and output (bottom) for an example test trial for each of the three training phases. **G-I.** Average absolute recurrent input (y-axis) and feedback input (x-axis) weights after training phase one (G), two (H), and three (I). **J-L.** Change in feedback weights during the three phases of initial training. Note that networks learnt to "push" a fraction of the feedback weights towards zero, a phenomenon that correlated with the disappearance of oscillatory motor output after phase two.

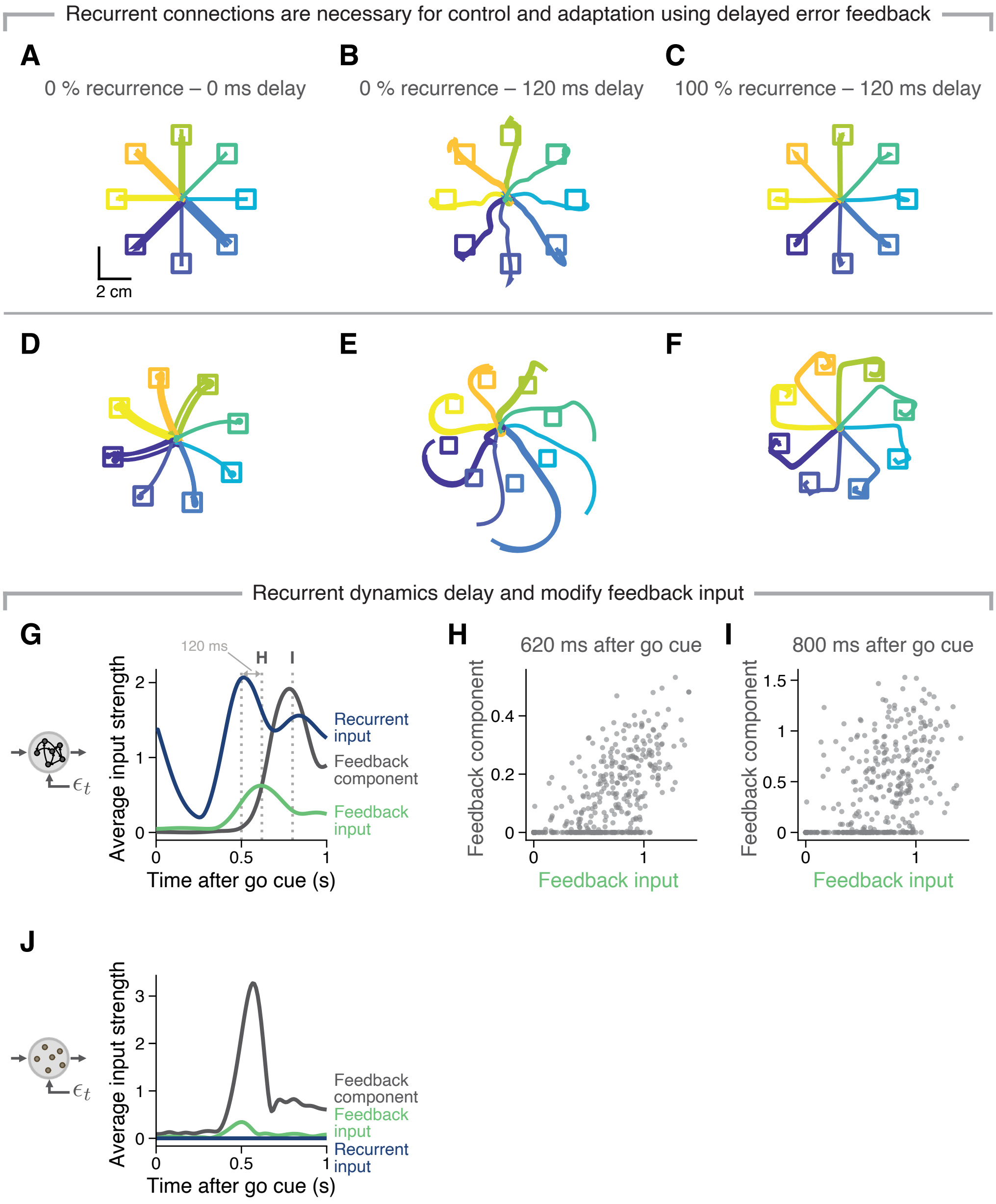

Supplementary Figure 3: Recurrent connectivity enables stable control with delayed feedback. Three different model variants are compared in A-F. Left (A,D), a network without any recurrent connections and with no time delay in the feedback signal. Middle (B,E), the same network without recurrent connections but with a time delayed feedback signal. Right (C,F), the default model used throughout the paper with recurrent connections and a time delayed feedback signal. **A-C.** Hand trajectories produced by the RNN. **D-F.** Hand trajectories after introducing a 30° rotation of the RNN's output, to mimic a visuomotor rotation perturbation. **G.** Average recurrent (blue) and feedback input (red), and average feedback response (black). The average feedback response is computed by taking the difference in neural activity between perturbed and unperturbed trials (cf. Figure 3). **H.** Feedback component against feedback input for single neurons 620ms after GO signal. **I.** Same as (J) but 800ms after GO signal. **J.** Same as (G) but for network without any recurrent connections.

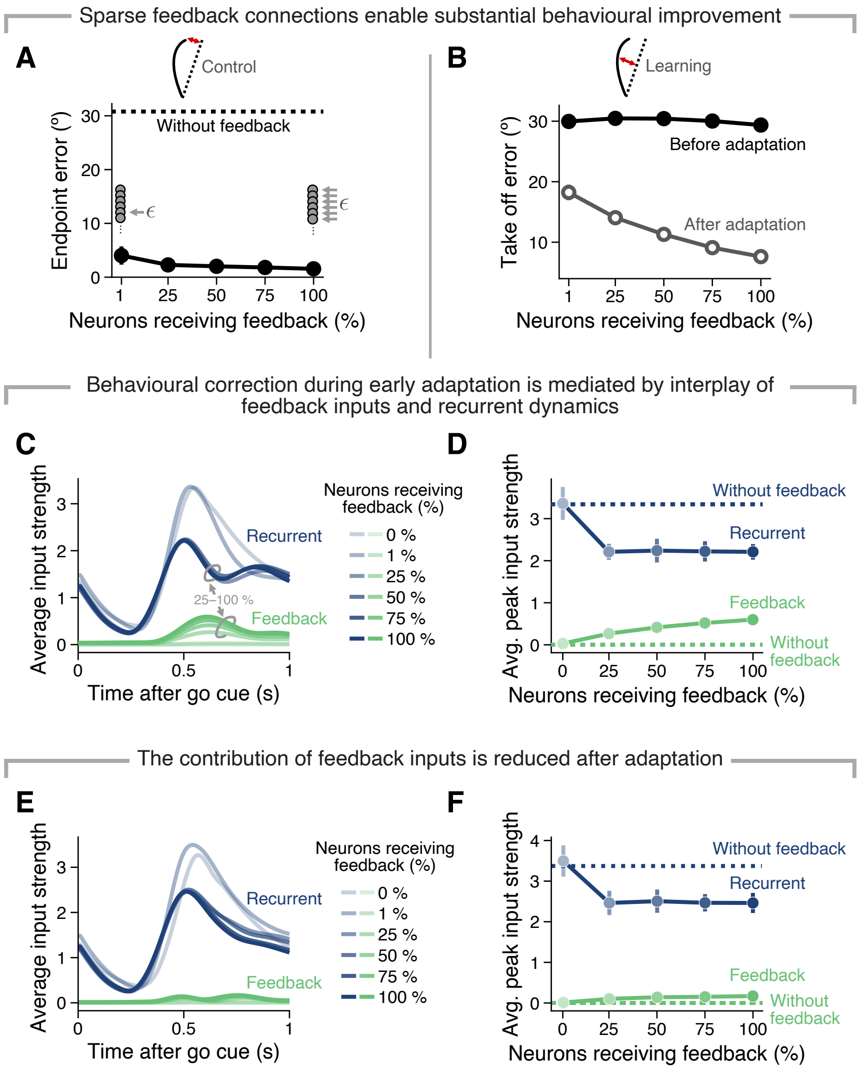

Supplementary Figure 4: Both feedback-based motor control and adaptation can be achieved with sparse feedback signals which are small compared to recurrent signals. A. Angular error between produced and target position at the end of the reach immediately after onset of visuomotor rotation for networks with different percentages of neurons receiving afferent feedback (black markers), including no feedback (dashed line). Lines and error bars, mean and s.d. across ten networks. **B.** Take-off error at visuomotor rotation onset (solid circles), and after adaptation (empty circles) for networks with different percentages of neurons receiving afferent feedback. Lines and error bars, mean and s.d. across ten networks. **C.** Average recurrent (blue) and feedback (red) inputs to an RNN neuron before adaptation. Average input strength is defined as the mean across incoming signals and neurons. Legend, percentage of neurons receiving feedback. **D.** Average magnitude of the peak input strength of the recurrent (blue) and feedback (red) inputs before adaptation. Same colour scheme as in C. Lines and error bars, mean and s.d. across ten networks. **E.** Average recurrent (blue) and feedback (red) inputs to an RNN neuron after adaptation. Data presented as in C. **F.** Average magnitude of the peak input strength of the recurrent (blue) and feedback (red) inputs after adaptation. Data presented as in D.

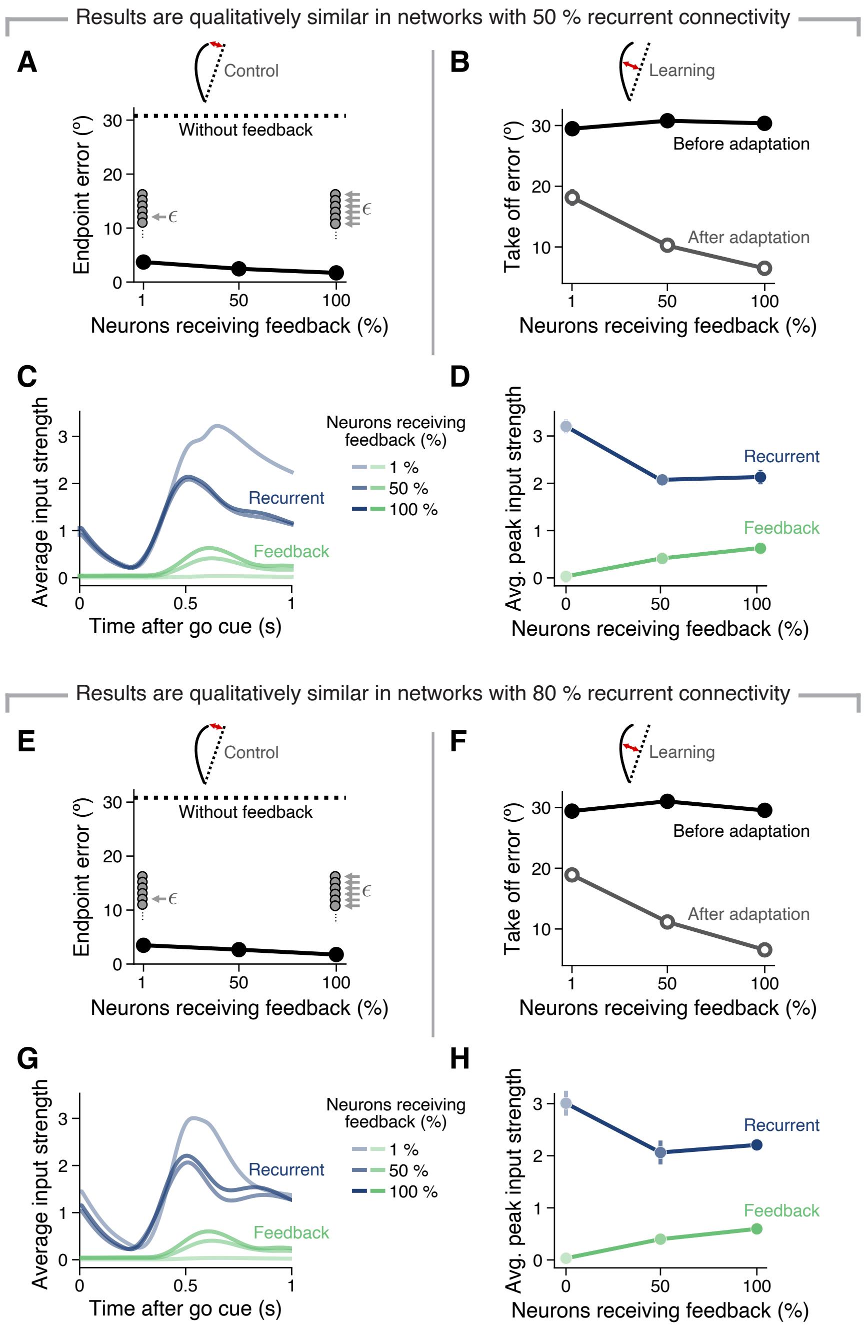

Supplementary Figure 5: Effective feedback-based motor control and adaptation can both be achieved with sparse feedback for varied degrees of recurrent connectivity. Simulation results for networks with 50% (**A-D**) or 80% (**E-H**) recurrent connection probability. **A.** Angular error between produced and target position at the end of the reach immediately after onset of visuomotor rotation onset for networks with different percentages of neurons receiving afferent feedback (black markers), including no feedback (dashed line). Lines and error bars, mean and s.d. across ten networks. **B.** Take-off error at visuomotor rotation onset (solid circles), and after adaptation (empty circles) for networks with different percentages of neurons receiving afferent feedback. Lines and error bars, mean and s.d. across ten networks. **C.** Average recurrent (blue) and feedback (red) inputs to an RNN neuron before adaptation. Average input strength is defined as the mean across incoming signals and neurons. Legend, percentage of neurons receiving feedback. **D.** Average magnitude of the peak input strength of the recurrent (blue) and feedback (red) inputs before adaptation. Same colour scheme as in C. Lines and error bars, mean and s.d. across ten networks. **E-H.** Same as A-D but for networks with 80% recurrent connection probability.

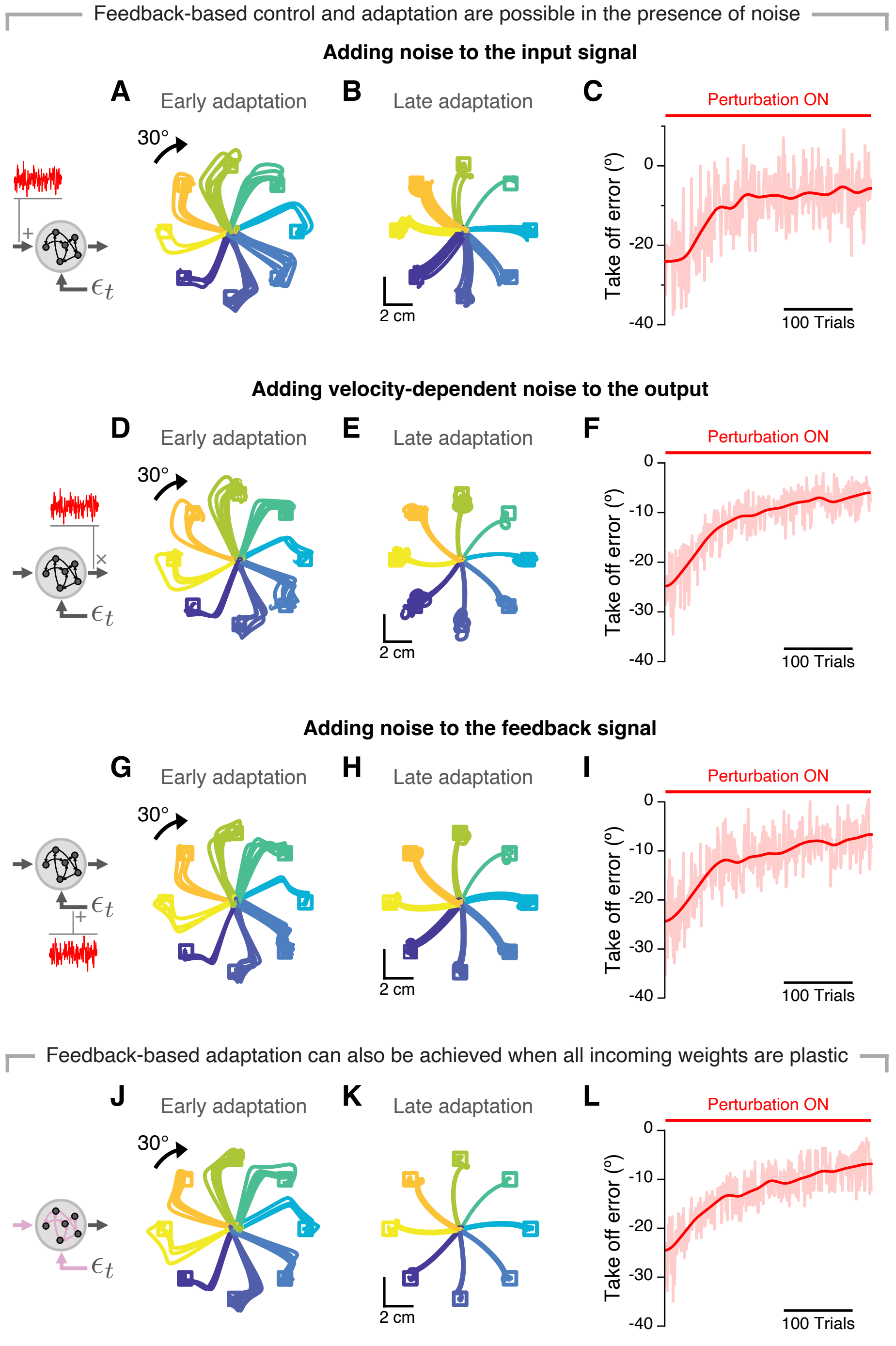

Supplementary Figure 6: Control and adaptation can be achieved with noisy signals and less localised plasticity rule. Here we present four different control simulations validating our main results. The first three rows correspond to noise added at different stages in the model, whereas the fourth row corresponds to adaptation with plasticity on all incoming weights of the network. Panel organization is identical in each row. Left, produced hand trajectories after a 30° rotation of the RNN's output. Middle, hand trajectories during late adaptation. Right, angular reaching take-off error for the perturbation phase. Transparent lines, single trial errors; solid lines, smoothed mean error (Gaussian filter, s.d., 10 trials). Left and middle panel show first and last 40 trials of that phase respectively. **A-C.** Learning in the presence of additive input noise. Gaussian random numbers ($\mu = 0.0$, $\sigma = 0.1$) are added for each trial and dimension of the input signal s. **D-F.** Learning in the presence of velocity-dependent noise applied to the output. To add multiplicative noise to the output of the RNN we first draw Gaussian random numbers for each trial, time point and dimension of the output signal v with ($\mu = 0.0$, $\sigma = 0.1$). Then we smooth it across time with a moving average kernel of length 50ms. The resulting values are multiplied with and added to the velocity output of the RNN. **G-I.** Learning in the presence of additive feedback noise. Gaussian random numbers ($\mu = 0.0$, $\sigma = 0.1$) are drawn for each trial, time point and dimension of the feedback signal ϵ. **J-L.** Learning with plasticity in all incoming weights (cf. Supplementary Methods).

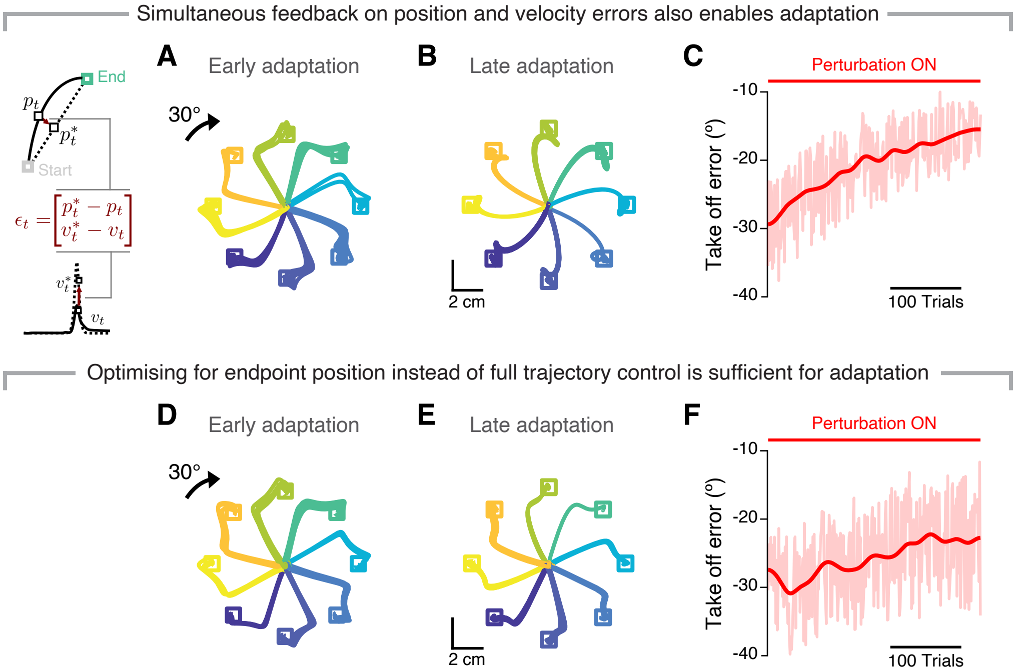

Supplementary Figure 7: Online motor control and feedback-based learning can be achieved for both higher-dimensional joint position and velocity feedback, and for end-point instead of trajectory control. Panel organization is identical in each row. Left, produced hand trajectories after a 30° rotation of the RNN's output. Middle, hand trajectories during late adaptation. Right, angular reaching take-off error for the perturbation phase. Transparent lines, single trial errors; solid lines, smoothed mean error (Gaussian filter, s.d., 10 trials). Left and middle panel show first and last 40 trials of that phase respectively. **A-C.** Higher-dimensional feedback signal. Here, the feedback signal ϵ does not only carry the positional error (default in the main paper), but also the velocity error. **D-F.** Endpoint instead of trajectory control. Here, we changed the loss function during the initial training phase to only take into account the first and the last 500 ms of each trial, thereby only optimizing the start and endpoint of each reach. During adaptation we adapt the plasticity rule such that it only takes into account the last 500 ms of each trial.

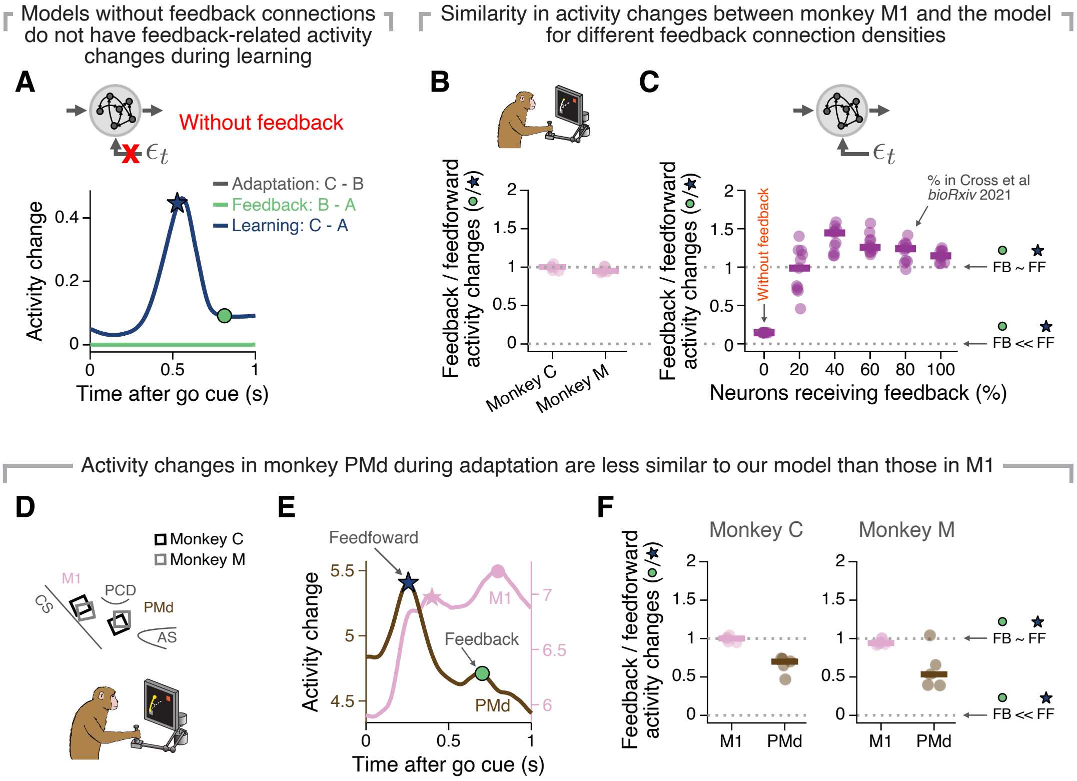

Supplementary Figure 8: Activity changes during VR for monkey PMd and M1, and additional data for Figure 3. A. Activity changes during VR in a model without feedback signal. Data presented as in Figure 3. **B-C.** Comparison between the ratio of the feedback-related activity changes (green circle in Figure 3C,D) to feedforward-related activity changes (blue star in Figure 3C,D) for monkey M1 (data shown for each monkey separately), and networks with different feedback projection densities (Panel **C**). Note how networks with dense projection densities (which best match electrophysiological observations[2]) show activity changes that are closest to those observed in the monkey data. Individual markers, individual experimental sessions or simulation runs; horizontal line, mean. FB, feedback; FF, feedforward. **D-F.** Neural activity changes in dorsal premotor cortex (PMd) of two monkeys during VR experiment. Data presented as in Figure 3. Note that activity changes in PMd look less like our networks' than M1 activity changes, probably due to PMd's less prominent feedback responses. The monkey image was created by Carolina Massumoto who gave permission to use it under CC-BY license.

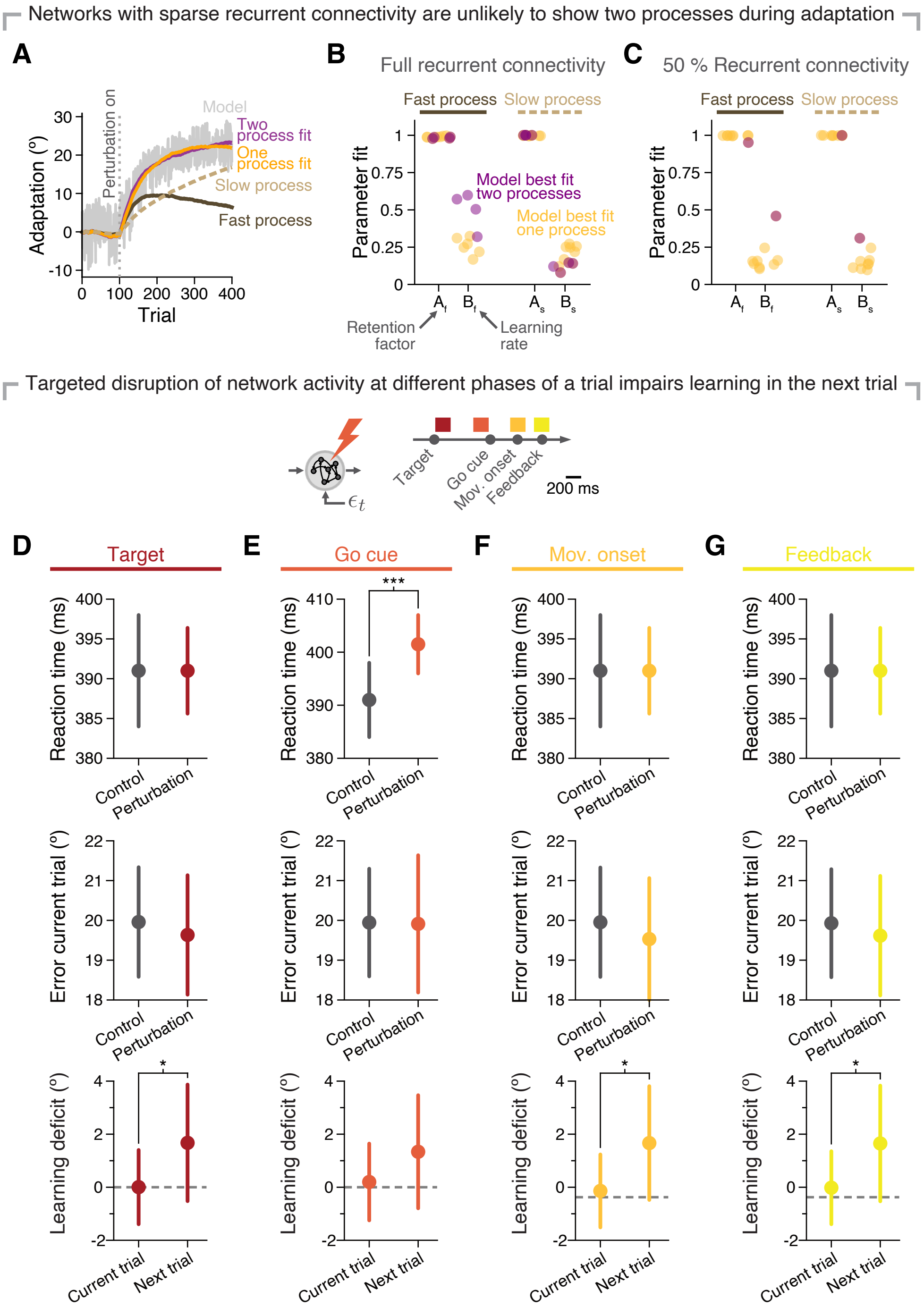

Supplementary Figure 9: Extended analyses for experiments presented in Figure 5. A-C. Multi-timescale learning. We test whether a two-state model fits the data significantly better than a one-state model (F-test). Purple circles, networks where the two-state model is significantly better. Yellow circles, networks where the two-state model is not significantly better. Data presented as in Figure 5. **D-G.** Perturbation to network activity at different time windows within a trial: around target presentation (D), before the GO cue (E), around movement onset (F), around feedback response (G). Data presented as in Figure 5.

Supplementary Methods

Plasticity rule on all incoming weights

$$
\begin{aligned}
d\tilde{W}_{ji}(t) &= \mathrm{dt}\ \eta \sum_{k=x,y} F_{jk}\epsilon_k(t-\Delta) r_i(t) \\
d\tilde{W}^{in}_{ji}(t) &= \mathrm{dt}\ \eta \sum_{k=x,y} F_{jk}\epsilon_k(t-\Delta) \sum_{t'<t}^{T} s_i(t') \\
d\tilde{F}_{ji}(t) &= \mathrm{dt}\ \eta \sum_{k=x,y} F_{jk}\epsilon_k(t-\Delta) \sum_{t'<t}^{T} \epsilon_i(t'-\Delta)
\end{aligned}
$$

where W is the recurrent weight matrix, W^{in} is the input weight matrix, and F is the feedback weight matrix (cf. detailed variable definition in main manuscript). The weight updates are performed as described in the main article.

References

[1] Paul Cisek. Internal Models. In Marc D. Binder, Nobutaka Hirokawa, and Uwe Windhorst, editors, *Encyclopedia of Neuroscience*, pages 2009–2012. Springer Berlin Heidelberg, Berlin, Heidelberg, 2009.

[2] Kevin P Cross, Douglas J Cook, and Stephen H Scott. Convergence of proprioceptive and visual feedback on neurons in primary motor cortex. *bioRxiv*, 2021.
