# Supplementary material for: A neural implementation model of feedback-based motor learning
Source: Nat Commun. 2025 Feb 20;16:1805. doi: 10.1038/s41467-024-54738-5 (PMC11842561; doi:10.1038/s41467-024-54738-5)
Supplement: Supplementary file 2 — Reporting Summary [file 41467_2024_54738_MOESM2_ESM.pdf]

nature portfolio

Corresponding author(s): Dr Juan Alvaro Gallego, Prof Claudia Clopath

Last updated by author(s): Oct 30, 2024

Reporting Summary

Nature Portfolio wishes to improve the reproducibility of the work that we publish. This form provides structure for consistency and transparency in reporting. For further information on Nature Portfolio policies, see our Editorial Policies and the Editorial Policy Checklist.

Statistics

For all statistical analyses, confirm that the following items are present in the figure legend, table legend, main text, or Methods section.

n/a	Confirmed	
☐	☒	The exact sample size (n) for each experimental group/condition, given as a discrete number and unit of measurement
☐	☒	A statement on whether measurements were taken from distinct samples or whether the same sample was measured repeatedly
☐	☒	The statistical test(s) used AND whether they are one- or two-sided<br>*Only common tests should be described solely by name; describe more complex techniques in the Methods section.*
☒	☐	A description of all covariates tested
☒	☐	A description of any assumptions or corrections, such as tests of normality and adjustment for multiple comparisons
☐	☒	A full description of the statistical parameters including central tendency (e.g. means) or other basic estimates (e.g. regression coefficient) AND variation (e.g. standard deviation) or associated estimates of uncertainty (e.g. confidence intervals)
☐	☒	For null hypothesis testing, the test statistic (e.g. F, t, r) with confidence intervals, effect sizes, degrees of freedom and P value noted<br>*Give P values as exact values whenever suitable.*
☒	☐	For Bayesian analysis, information on the choice of priors and Markov chain Monte Carlo settings
☒	☐	For hierarchical and complex designs, identification of the appropriate level for tests and full reporting of outcomes
☒	☐	Estimates of effect sizes (e.g. Cohen's d, Pearson's r), indicating how they were calculated

Our web collection on statistics for biologists contains articles on many of the points above.

Software and code

Policy information about availability of computer code

Data collection	Custom simulation code was written in Python-3.7.4 using the pytorch-1.8.1 package to train the neural network models. The code will be made publicly available upon publication under https://github.com/babaf/feedback-driven-plasticity
Data analysis	All data analysis was performed using Python-3.7.4.

For manuscripts utilizing custom algorithms or software that are central to the research but not yet described in published literature, software must be made available to editors and reviewers. We strongly encourage code deposition in a community repository (e.g. GitHub). See the Nature Portfolio guidelines for submitting code & software for further information.

Data

Policy information about availability of data

All manuscripts must include a data availability statement. This statement should provide the following information, where applicable:

- Accession codes, unique identifiers, or web links for publicly available datasets
- A description of any restrictions on data availability
- For clinical datasets or third party data, please ensure that the statement adheres to our policy

The electrophysiology and behavioural data supporting the findings from this modeling study will be made available from the senior author upon request.

Research involving human participants, their data, or biological material

Policy information about studies with human participants or human data. See also policy information about sex, gender (identity/presentation), and sexual orientation and race, ethnicity and racism.

Reporting on sex and gender	N/A
Reporting on race, ethnicity, or other socially relevant groupings	N/A
Population characteristics	N/A
Recruitment	N/A
Ethics oversight	N/A

Note that full information on the approval of the study protocol must also be provided in the manuscript.

Field-specific reporting

Please select the one below that is the best fit for your research. If you are not sure, read the appropriate sections before making your selection.

- [x] Life sciences
- [ ] Behavioural & social sciences
- [ ] Ecological, evolutionary & environmental sciences

For a reference copy of the document with all sections, see nature.com/documents/nr-reporting-summary-flat.pdf

Life sciences study design

All studies must disclose on these points even when the disclosure is negative.

Sample size	To compare activity changes in our models to activity changes in actual brains, we analysed data from four implants from two different monkeys to show reproducibility. Given our recording methods, we could not control the number of neurons studied for these analyses, but we recorded from sufficient neurons to extract information about the population. For example, application of Principal Component Analysis to the binned firing rates identified a few (e.g., 6-10) dominant patterns of neural covariation that explained a substantial amount of total neural variance (typically >50 %). For all model simulations, we always simulated ten neural networks to ensure reproducibility across network initialisations.
Data exclusions	For the electrophysiology data, since we used extracellular recordings collected using chronically implanted microelectrode arrays, we could not control the specific cells we recorded from. Thus, we excluded some cells with low firing rates (<1 Hz mean firing rate across all time bins) or very poor recording quality. The latter was assessed using a combination of: 1) visual identification of threshold crossing waveforms that appeared to be artifacts rather than from neural origin, and 2) an analysis of cross-channel 'shunting' based on calculating cross-correlograms of the binned neural firing rates computed in very small bins (1 ms). No data were excluded from the simulation analyses.
Replication	We ran several control simulations (cf. Supplementary figures) that all qualitatively supported the main findings in the paper. For the electrophysiology data, we reproduced our main findings in 11 sessions from two different monkeys.
Randomization	This allocation was not relevant for this study, since we studied all experimental subjects under similar behavioural conditions. There are no subjects or groups across the models.
Blinding	Blinding was not relevant to this study since, e.g., we did not seek to compare the outcomes of different interventions. There are no subjects or groups across the models.

Reporting for specific materials, systems and methods

We require information from authors about some types of materials, experimental systems and methods used in many studies. Here, indicate whether each material, system or method listed is relevant to your study. If you are not sure if a list item applies to your research, read the appropriate section before selecting a response.

Materials & experimental systems

n/a	Involved in the study	
☒	☐	Antibodies
☒	☐	Eukaryotic cell lines
☒	☐	Palaeontology and archaeology
☐	☒	Animals and other organisms
☒	☐	Clinical data
☒	☐	Dual use research of concern
☒	☐	Plants

Methods

n/a	Involved in the study	
☒	☐	ChIP-seq
☒	☐	Flow cytometry
☒	☐	MRI-based neuroimaging

Animals and other research organisms

Policy information about studies involving animals; ARRIVE guidelines recommended for reporting animal research, and Sex and Gender in Research

Laboratory animals	This study used data from two macaque monkeys (both male, macaca mulatta). Monkey C was eight years old at the time of the recordings, and monkey M was six-seven years old.
Wild animals	This study did not involve wild animals.
Reporting on sex	Sex was not considered in the study; both monkeys were male.
Field-collected samples	This study did not involve samples collected in the field.
Ethics oversight	All surgical and experimental procedures were approved by the Institutional Animal Care and Use Committee (IACUC) of Northwestern University

Note that full information on the approval of the study protocol must also be provided in the manuscript.

Plants

Seed stocks	N/A
Novel plant genotypes	N/A
Authentication	N/A
